# Neurodevelopment at 11 months after starting antiretroviral therapy within 3 weeks of life

**DOI:** 10.4102/sajhivmed.v20i1.1008

**Published:** 2019-10-30

**Authors:** Barbara Laughton, Shalena Naidoo, Els F.M.T. Dobbels, Michael J. Boivin, Anita Janse van Rensburg, Richard H. Glashoff, Gert U. van Zyl, Mariana Kruger, Mark F. Cotton

**Affiliations:** 1 Department of Paediatrics and Child Health, Stellenbosch University, Cape Town, South Africa; 2 Department of Pathology, Stellenbosch University, Cape Town, South Africa; 3 Department of Psychiatry, Michigan State University, East Lansing, United States; 4 Department of Neurology and Ophthalmology, Michigan State University, East Lansing, United States; 5 National Laboratory Services, Cape Town, South Africa; 6 Division of Virology, Faculty of Medicine and Health Sciences, Stellenbosch University, South Africa

**Keywords:** HIV, infants, antiretroviral therapy, neurodevelopment, paediatrics

## Abstract

**Background:**

Antiretroviral therapy (ART) started between 7 and 12 weeks of age improves neurodevelopmental outcomes in HIV-infected (HIV+) infants, but the impact of even earlier initiation is not yet described.

**Objectives:**

We assessed the early neurodevelopment of HIV+ infants who started ART within 21 days of life.

**Method:**

Participants were enrolled from the public sector birth HIV-diagnosis programme. Inclusion criteria included the following: birth weight > 2000 g, infant commencing ART < 6 weeks and no infant cytomegalovirus disease. Antiretroviral therapy included Zidovudine/Lamivudine/Nevirapine for the first 2 weeks, the latter then replaced by Lopinavir/Ritonavir. Once body weight > 3 kg and gestational age > 44 weeks, Abacavir replaced Zidovudine. The Griffiths mental development scales (GMDS) were administered at 10–12 months.

**Results:**

Of 29 infants assessed, 23 (79%) were girls. Mean birth weight was 3002 ± 501 g. Twenty-four mothers (83%) received ART during pregnancy. Seven (24%) infants were diagnosed HIV+ within 48 h of birth. Median [interquartile range] viral load (VL) at diagnosis was 3904 [259–16 922] copies/mL, age starting ART was 6.0 [3–10] days and age at VL suppression was 19.1 [15–36] weeks. At the GMDS assessment, nine (31%) participants had detectable VL and 26 (90%) had World Health Organization (WHO) clinical stage I disease. The GMDS was performed at a mean age of 11.5 ± 0.8 months. Mean quotients were within the average range: Global Griffiths score was 103.6 ± 10.9 and mean quotients on the subscales ranged from lowest 95.9 ± 13.4 for locomotor to highest 112.8 ± 11.3 for hearing-and-language.

**Conclusion:**

Preliminary findings in this small group suggest that early neurodevelopmental scores are within the normal range in infants with perinatal HIV infection who started ART at a median of 6 days.

## Introduction

HIV-infected (HIV+) children are at risk for neurodevelopmental delay. Neurologic insults develop after HIV enters the brain, creating an inflammatory state affecting neuronal and astrocyte growth and development^1^; the most severe manifestation being HIV encephalopathy (HIVE). HIV encephalopathy rates range from 6% to 30%, with higher rates in low- and middle-income countries especially with delayed initiation of combination antiretroviral therapy (ART).^2,3,4,5^ These consequences place a great burden on the social and healthcare systems.

Antiretroviral therapy has decreased the incidence of HIVE,^6^ and when initiated between 3 and 9 months of age, it improves clinical and neurodevelopmental outcomes.^7,8,9,10^ Nevertheless, studies describing permanent deficits or lack of catch-up suggest that prevention is better than reversal of HIVE.^11,12,13^ From 2016, the World Health Organization (WHO) introduced birth testing and recommended starting ART as soon as possible once HIV infection is confirmed.^14^ Since the report of temporary remission in the Mississippi baby after very early ART,^15^ there is increasing evidence that early ART in perinatally infected children improves infant outcomes.^16^ Early ART can limit HIV reservoir size, and when started before 2 months of age, it is associated with fewer infected and transcriptionally active cells and less infectious virus recovery.^17,18,19,20,21,22,23,24^ However, administering ART in a neonate and young infant is not easy with potential drug resistance because of under-dosing, or neurotoxicity because of overdosing.^25^ The long-term outcomes of very early exposure to ART are still unknown. It is therefore imperative that neurodevelopmental testing be undertaken after early ART initiation. Our aim was to determine the neurodevelopmental outcomes of perinatally HIV-infected children after initiating ART within the first 3 weeks of life.

## Research methods and design

We report early data from a prospective descriptive study conducted in the Family Centre for Research with Ubuntu (FAM-CRU) in Tygerberg Hospital, Cape Town, South Africa, with recruitment from the Médecins Sans Frontières service in Khayelitsha and elsewhere in the public sector. Antiretroviral therapy was started as soon as HIV infection was confirmed. HIV diagnosis was made by quantitative HIV-1 viral load (VL) testing and confirmed by a qualitative HIV-1 RNA PCR. Indeterminate samples were repeated until HIV diagnosis confirmation.^26,27^ Inclusion criteria were the following: birth weight > 2000 g, commencing ART < 6 weeks of age and no infant cytomegalovirus (CMV) infection. Mothers or legal guardians were consented in person in their language of choice according to Good Clinical Practice standards.

Participants were seen as frequently as needed until stable, monthly for 3 months and then 3 monthly. Visits included a medical examination, growth monitoring, adverse event assessment and social work support where needed. At each visit, a pharmacist calculated the percentage adherence for each drug from returned ART containers and an adherence counsellor established reasons for over or under-dosing with the parent or caregiver, offered advice on problems identified and reviewed measuring techniques. HIV viral load was performed at baseline, 3, 6 and 12 months. Undetectable VLs were reported as < 100 or < 40 copies/mL depending on the blood volume available for testing. CD4 cell counts were done at 3, 6 and 12 months. Antiretroviral therapy comprised Zidovudine, Lamivudine and Nevirapine, with Lopinavir/Ritonavir replacing Nevirapine after 2 weeks of age or gestational age of 42 weeks. Once weight exceeded 3 kg and gestational age was above 44 weeks, Abacavir replaced Zidovudine. Participants also received co-trimoxazole from 6 weeks of age.

The Griffiths mental development scales (GMDS) (0–2 years) were conducted by the same developmental paediatrician (B.L.) at 10–12 months of age.^28^ The GMDS assesses five subscales: locomotor, personal–social, hearing-and-language, eye–hand coordination and performance (visual–motor abilities). A global score, the General Griffiths, is also calculated. Raw scores are converted into quotients, derived from norms of healthy British children, with a mean of 100 and standard deviation (s.d.) of 16. While the GMDS is neither standardised nor validated in South Africa, it is the most widely used developmental assessment tool, is considered culturally fair and is used to assess young children including those exposed to HIV.^29,30,31,32,33,34,35,36^ Vision was assessed clinically during testing and through the ability to track small cake decorations (‘hundreds and thousands’ test), which implies visual acuity of 6/24 or better.^37^

Statistical analysis was performed using Stata release 11 (StataCorp, College Station, TX) and Statistica 13 (software.dell.com. Dell Inc. 2015). For descriptive statistics, mean and s.d. were reported for normally distributed data and median and interquartile range (IQR) for skewed data. Guided by distribution of the data, Spearman and Pearson correlations were used to explore correlation between various parameters and neurodevelopmental outcomes. For calculating age at VL suppression, those who had not yet achieved VL suppression were assigned a date 2 days after the GMDS. Regression analysis explored the contribution of five predictors of GMDS scores: birth weight, ART start age, baseline VL, baseline CD4% and age at first VL suppression.

Descriptive data and GMDS scores were also compared to those from the early treatment arms on Children with HIV Early antiRetroviral treatment (CHER) trial participating in a neurodevelopmental sub-study who received early ART from a median of age of 7.7 weeks and were assessed by the same investigators at 11 months of age.^10^

### Ethical considerations

Mothers or legal guardians were consented in person in their language of choice according to Good Clinical Practice standards. The Stellenbosch University Health Research Ethics Committee approved the study (No.: M14/07/029).

## Results

Of 29 children studied, 23 (79%) were female. Mean birth weight was 3002 ± 501 g and gestation was 37.9 ± 2.3 weeks. HIV+ diagnosis was made by 48 h of birth in 7 (24%) and within 7 days of birth in 17 (59%) infants. Median [IQR] age for starting ART was 6.0 [3–10] days (range 0–21) from birth. Twenty-three achieved VL suppression at median [IQR] 19.1 [14.7–35.9] weeks of age (range 2–53) (Table 1).

**TABLE 1:** Demographic characteristics of participants (*N* = 29).

Characteristics	Result
Sex	Female = 23 (79%)
Birth weight (g) Mean ± s.d.	3002 ± 501 (2150–4070)
Gestational age (weeks) Mean ± s.d. (range) (3 unknown gestation)	37.9 ± 2.3 (33–41)
Birth method: Vertex delivery Caesarean section	21 (72%) 8
Mother’s age at birth (years) Mean ± s.d. (range)	29.3 ± 5.4 (18.9–40.4)
History of prenatal substance exposure	2 – Methamphetamine 1 – Alcohol + methamphetamine
Home language	21 (70%) Xhosa 6 (21%) Afrikaans 1 Shona 1 English
PMTCT – mother	24 (83%) Yes 4 (14%) No 1 Unknown
Infant age HIV diagnosis (days) Median [IQR] (range)	6 [3–12] (0–52)
Infant age HIV diagnosis: within 48 h within 1 week	7 (24%) 17 (59%)
Infant ART start age (days) Median [IQR] (range)	6.0 [3–10] (0–21)
ART regimen started *n* (%)	16 (55%) Zidovudine, Lamivudine, Nevirapine 6 (20%) Zidovudine, Lamivudine, Lopinavir/Ritonavir 7 (24%) Abacavir, Lamivudine, Lopinavir/Ritonavir
Infant baseline VL (copies/mL) Median [IQR] (*n*=26†)	3904 [265–16 922] (range 99–201 916)
CD4 closest to baseline Median [IQR] Age (days) Absolute count CD4%	14 [9–28] (range 0–251‡) 1938 [1446–2570] (range 679–3776) 43 [35–56] (range 19.6–71)
Time to undetectable VL§ (weeks from birth) Median [IQR] (range)	19.1 [15–34] (2–53) (*n* = 23¶)

VL, viral load; IQR, interquartile range; ART, Antiretroviral therapy; PMTCT, prevention of mother-to-child transmission; s.d., standard deviation; GMDS, Griffiths Mental Development Scale.

† , Three only had HIV PCR+ and no VL measured.

‡ , Participant only enrolled into study at this age – only had VL before and no CD4 counts.

§ , VL done at baseline/diagnosis, 3, 6, 12 and 18 months.

¶ , Six did not suppress by time of GMDS assessment.

The GMDS was performed at a mean of 11.5 ± 0.8 months (range 10.2–13.1) and scores are described in Table 2. Mean GMDS quotients were in the average range and within 1 s.d. of the standardised scores. The locomotor subscale had the lowest mean quotient. No children were suspected of having hearing or vision problems.

**TABLE 2:** Scores on Griffiths mental development scales (quotients) at mean age of 11.5 months (*n* = 29).

Scale	Mean	Standard deviation	Maximum	Minimum
Locomotor	95.9	13.4	125	74
Personal–social	104.2	14.7	138	72
Speech and hearing	112.8	11.3	131	85
Eye–hand coordination	105.0	17.5	136	60
Performance (visual–spatial)	99.1	15.7	133	68
General Griffiths	103.6	10.9	123	82

Note: Norms from healthy British children: mean 100 ± 16.

Clinical status at the time of GMDS is described in Table 3. One child had progressed to WHO stage II HIV disease (persistent oral candida), and two to stage III (chronic suppurative otitis media and pulmonary tuberculosis). Nine children (31%) had detectable VL at the time of GMDS testing, six (21%) had not yet achieved viral suppression and three had previously suppressed (one at 27 weeks and two at 19 weeks of age), but rebounded to log 5.44 (273 328 copies/mL), log 3.18 (1519 copies/mL) and log 4.46 (28 649 copies/mL), respectively. Another participant suppressed at 3 months, and had a viral blip to 118 copies/mL at 6 months, with the VL undetectable 6 months later at GMDS.

**TABLE 3:** Clinical status at the time of neurodevelopmental assessments (*N* = 29).

Clinical characteristics	Result
Age: Mean ± s.d. (range)	11.5 ± 0.8 months (range 10.2–13.1)
HIV disease severity: WHO categories	1 = 26 (90%) 2 = 1 3 = 2
Growth: WHO *z*-scores for age (mean ± s.d.) Weight Length Head circumference	−0.09 ± 0.9 (range −1.7 to 1.6) −1.1 ± 1 (range −3.5 to 0.2) 0.23 ± 1 (range −1.9 to 2.4)
ART regimen	29 Abacavir, Lamivudine, Lopinavir/Ritonavir
VL undetectable, *n* (%)	20 (69%)
CD4 closest to GMDS Median [IQR] Absolute count CD4%	2097.9 [743–1512] (range 863–3790) 33.8 [27–41] (range 18–53)
CD8 closest to GMDS Median [IQR] Absolute count CD8%	1489 [1131–2437] (range 608–7551) 27 [21–34] (range 13–53)
CD4/CD8 ratio closest to GMDS Median [IQR]	1.33 [0.83–1.92] (0.38–3.79)
Current caregiver *n* (%)	23 (79%) mother 1 shared mother and grandmother 2 aunt 1 foster mother 2 grandmother
Father or father-figure present, *n* (%) Caregiver/father/father-figure Drugs or alcohol abuse, *n* (%) Housing, *n* (%): Brick Informal dwelling Electricity in house, *n* (%) Household receives social grants, *n* (%)	20 (69%) 7 (24%) 10 (34%) 19 (66%) 28 (97%) 26 (90%)

IQR, interquartile range; s.d., standard deviation; GMDS, Griffiths Mental Development Scale.

A number of demographic and exposure issues with potential to influence neurodevelopmental outcomes were identified. These included two without antenatal care, one with an unsupervised home birth and three with maternal substance abuse: two methamphetamine and one methamphetamine and alcohol (over time these children were fostered by caring relatives). Medical problems included one each of the following: congenital pneumonia of unknown aetiology, intrauterine growth retardation, neonatal jaundice above exchange transfusion levels (resolved without exchange), congenital syphilis with mild hypoxia and suspected seizure, mild birth asphyxia (low birth Apgar scores and cord blood pH = 7.17) and suspected hypoglycaemia (but glucose level not recorded) (these data not shown in any table).

The following adverse events, which could negatively impact neurodevelopment, were documented before the GMDS assessment: six with otitis media (one had two episodes), six with anaemia and three with neutropenia (Zidovudine was discontinued). One infant recovered fully after treatment for suspected bacterial meningitis and another was hospitalised for 6 months with pulmonary tuberculosis. Lastly, failure to thrive because of poor feeding and insufficient caloric intake occurred in one infant.

Adherence was calculated at a median [IQR] of 10 [9–11] visits. Only one participant had acceptable adherence percentages for all drugs at all visits. Three participants had poor adherence for more than half of the visits, with the rest over or under-dosing at various times. For the former, the infant would spit syrups out and caregivers were unsure how much to replace. For the latter, the caregivers either measured syrups incorrectly or were non-compliant. This prompted clinicians to encourage treatment supporters for the caregivers.

Correlations between GMDS scores and possible predictors of developmental outcomes (birth weight, gestation, maternal age, baseline VL, age starting ART, time to suppression and CD4 parameters at baseline) were not significant. The five predictors of GMDS scores entered into the regression model also did not show significant relationships, that is, birth weight, ART start age, baseline VL, baseline CD4% and age at first suppression. CD8 count at the time of GMDS showed a negative correlation with personal–social (Pearson *r* = −0.41; *p* = 0.03) and a negative trend with General Griffiths (Pearson *r* = −0.6; *p* = 0.06).

For growth parameters closest to the GMDS assessment, head circumference *z*-scores correlated significantly with the performance (visual–spatial) scores (Pearson’s *r* = 0.4; *p* = 0.02) and weight *z*-score correlated with eye–hand coordination scores (Pearson’s *r* = 0.36; *p* = 0.05). There was a positive trend between weight for age *z*-score and General Griffiths score (Pearson’s *r* = 0.34; *p* = 0.07).

We compared the GMDS scores of those whose mothers had ART for prevention of mother-to-child transmission of HIV (PMTCT) and those who did not and found no difference between the groups (see Appendix 1, Table 1-A1). There were also no significant differences on the GMDS scores between those with detectable VL and undetectable VL at the time of the test, despite the mean scores in the hearing-and-language and eye–hand coordination subtests being 5 points lower for the nine with detectable VL compared to the 20 with undetectable VL at testing (Table 4). We also compared the following participant demographics between the detectable VL and undetectable VL groups: birth weight, baseline VL copies and CD4 parameters, ART start age, CD4, CD8 and growth parameters at the time of GMDS, and found no difference (see Appendix 1, Table 2-A1).

**TABLE 4:** Comparison of Griffiths Mental Development Scale quotients in those with and without virological suppression at testing.

Viral load at testing	Detectable VL *n* = 9	Undetectable VL *n* = 20	p*
Mean age at testing (months)	11.4	11.5	
Locomotor	96.9 ± 13.5	95.4 ± 13.7	0.65
Personal–social	102.2 ± 13.9	105.1 ± 15.2	0.48
Hearing-and-language	109.2.4 ± 10.2	114.4 ± 11.6	0.32
Eye–hand coordination	101 ± 18.4	106.9 ± 17.3	0.46
Performance (visual–spatial)	97.9 ± 14.9	99.1 ± 16.4	0.94
General Griffiths	101.8 ± 10.4	104.4 ± 11.3	0.52

VL, viral load.

* , Mann–Whitney *U*.

The GMDS scores achieved by this cohort were similar to those from the CHER cohort (children on ART commenced at 7 weeks of age) at a mean age of 11.3 months,^10^ apart from personal–social subscale, where the CHER cohort had mean quotients 7 points above that of the current study population (Table 5). Post hoc item comparison for personal–social showed that CHER participants were more likely to help with dressing, hold an open cup for drinking, try to use a spoon for feeding and obey simple requests. Participants on the current study were more likely to clap hands and enjoy an adult showing a book.

**TABLE 5:** Comparison between study participants and Children with HIV Early antiRetroviral early treatment participants.^10^

Characteristics	Current study	CHER early ART	*p*
Number enrolled	29	64	-
Age of ART initiation Median [IQR]	6.0 [3–10] days	7.7 [7.1–9.5] weeks	< 0.001
Birth weight (g)	3002 ± 501	2994 ± 406	0.98
Gestational age (weeks)	37.9 ± 2.3 (3 unknown)	38.9 ± 2.3 (3 unknown)	0.06
PMTCT – mother Yes No Unknown	24 (83%) 4 (14%) 1 (3%)	55 (86%) 6 (9%) 3 (5%)	-
History of prenatal substance exposure	2 Methamphetamine 1 Alcohol + methamphetamine	2 Alcohol	-
Infant baseline VL (copies/mL)	2494 ± 47629 (*n* = 26)	5 500 942 ± 55 693	< 0.01†
CD4 absolute count	2090 ± 800	2062 ± 1100	0.42
CD4%	44.7 ± 14.2	35.2 ± 8.6	< 0.01
Time to undetectable VL‡ (weeks)	30.0 ± 16.6 (*n* = 23)	38.8 ± 8.8	0.01
ART regimen at the time of test	28 Abacavir, Lamivudine, Lopinavir/Ritonavir 1 Abacavir, Lamivudine, Didanosine	63 Lamivudine, Lopinavir/Ritonavir, Zidovudine 1 Abacavir, Nevirapine, Didanosine	-
VL undetectable at the time of GMDS test	20 (69%)	40 (62%)	-
Age at GMDS (months)	11.5 ± 0.8	11.3 ± 1.1	0.16
GMDS quotient scores:			
Locomotor	95.9 ± 13.4	97.7 ± 12.5	0.3
Personal–social	104.2 ± 14.7	111.2 ± 13.5	0.04
Speech and hearing	112.8 ± 11.3	112.5 ± 10.4	0.89
Eye–hand coordination	105.0 ± 17.5	107.4 ± 15.8	0.66
Performance (visual–spatial)	99.1 ± 16.1	100.3 ± 13.1	0.4
General Griffiths	103.6 ± 11.0	106.2 ± 10.4	0.21

Note: Norms from healthy British children: mean 100 ± 16. Results expressed as mean ± s.d.

VL, viral load; IQR, interquartile range; ART, Antiretroviral therapy; PMTCT, prevention of mother-to-child transmission; s.d., standard deviation; GMDS, Griffiths Mental Development Scale; CHER, Children with HIV Early antiRetroviral.

† , Mann–Whitney *U*.

‡ , For those not yet suppressed at assessment, date for suppression was allocated 2 days after assessment date.

Significant differences between the two groups are shown in Table 5, with CHER having higher VLs and lower CD4 counts at baseline and a longer time to undetectable VL compared to participants in the current study. Abacavir also replaces Zidovudine use in the CHER participants.

## Discussion

These findings from the first 29 infants who started ART at a median age of 6 days are encouraging and show potential for normal neurodevelopmental outcomes, despite other medical conditions in nine infants that may impair neurological development. These scores are well within 1 s.d. of the UK norms, and are comparable to other South African infants assessed at similar ages using the GMDS^29,30,32,33,34^ (see Appendix 1, Table 3-A1 for summary of scores). This finding is despite almost a third not being virologically suppressed at testing. However, VLs in this cohort indicated low exposure to HIV because of maternal ART.^38^

We previously described neurodevelopmental outcomes in the CHER trial at 11 months.^10^ We compared children on delayed ART to those who started early ART at a median [IQR] of 7.7 [7.1–9.5] weeks. The GMDS scores from the CHER early treatment arms are comparable to this very early treatment group, apart from the personal–social subscale (Table 5), which is the most subjective as caregiver report items are used, and may reflect a change in child-rearing practices over time with less emphasis on self-care skills. The CHER early treatment arms had a mean baseline VL of log_10_ copies/mL 5.64 which is far higher than the current study and baseline mean CD percentage of 35% which is lower than the current study, and longer time to undetectable VL. This may suggest that there is a safe window period for starting ART – between birth and a median of 7.7 weeks; however, these are early neurodevelopmental outcomes. Alternatively, were it not for adverse *in utero* exposures and non-suppressed VLs in six infants, the scores may have been higher than CHER early treatment participants. The early diagnosis of HIV+ infants within 48 h in 24% and by 7 days of age in 59% reflects high proportion of prenatal HIV infection, which also negatively impacts outcomes. In the CHER trial, *in utero* infection could not be assessed as infant screening began at 4–6 weeks of age for HIV.

An important finding is that we identified a number of challenges within the context of perinatal HIV infection, despite good PMTCT programmes. In those perinatally infected infants, a number of secondary effects, including systemic illnesses and environmental affects, may negatively impact a child’s early neurodevelopment.^39,40,41^ In our sample, we identified three with no prenatal care, three substance abuse, two congenital infections (syphilis and pneumonia of unknown aetiology), one co-infected with tuberculosis and one nutritional failure. Growth in participants was appropriate for weight and head circumference, but mean length *z*-score was −1.1.

We noted variability of ART adherence and the delay in attaining competence with ART dosing and adherence, with six children not yet suppressed at the time of GMDS assessment. Management of these young children was challenging as caregivers were non-compliant, under-skilled and found difficulty administering liquid formulations. Solid or dispersible formulations would certainly improve adherence.^42,43^ Our findings do not suggest neurotoxicity from ART.

This work had some limitations. As multiple factors may influence outcomes, 29 children starting ART very early are too few to assess weak associations with neurodevelopmental outcomes, including our finding of lower locomotor scores compared to other subscales. More girls than boys were enrolled in the sample; although previously described,^44^ this may be because of small sample size. We were not able to determine reliable predictors for neurodevelopmental outcomes, or compare the outcomes of suppressed and unsuppressed participants. This was also hindered by time to suppression being inaccurate as VLs were only done at baseline 3, 6 and 12 months. We did not collect information on maternal health, immune status, VL or antiretroviral therapies. In the absence of South African normative data on the GMDS, a control or comparison group would have been helpful. However, we have experience in this community using the GMDS and are able to use these results for comparison^30,32^ (Appendix 1, Table 3-A1). The confounding problems of mothers with substance abuse did not seem to have a major impact, but the limitation is probably sample size.

Our findings are relevant to upscaling neonatal HIV identification and care.^45,46^ While the number of HIV+ infants is decreasing, this population remains at high risk because of structural and behavioural challenges in providing appropriate care. As liquid Lopinavir/Ritonavir formulation is poorly tolerated, newer formulations and other alternatives such as integrase inhibitors will be better accepted. Healthcare planners should not downscale programmes according to decreasing numbers, as those failing PMTCT require a higher level of care and intensive intervention to enable benefit from early ART. With the potential of early ART to limit HIV reservoir seeding, and potential to contribute to functional cures, treatment programmes need to support these vulnerable infants and their caregivers.^47^ Mentor mothers as treatment supporters may decrease the burden of HIV care and consequences of developmental delay, and could be very important when planning programmes. If these needs can be met, our findings are encouraging.^48,49^

## Conclusion

Preliminary findings in this small group suggest that despite PMTCT failure, children infected perinatally with HIV may have typical neurodevelopment if starting ART at a median age of 6 days, and similar to those starting ART at a median of 7 weeks. Good supportive care, including for ART adherence, is essential. A larger cohort that includes controls is in study and the findings at 18 months of age will inform on the influence of time to VL suppression and reservoir size and also the influence of social factors and demographic factors on neurodevelopmental outcomes. This may also allow for more precise study of locomotor outcomes.

## Appendix 1

**TABLE 1-A1:** Comparison of Griffiths mental development scales quotients in those whose mothers had antiretroviral therapy for prevention of mother-to-child transmission of HIV or not reported as mean (standard deviation).

Scale	Yes PMTCT *N* = 24	No PMTCT *N* = 4	*p*
Locomotor	96.9 (14)	92 (15)	0.51
Personal–social	104.0 (15)	111.8 (6)	0.22
Hearing-and-language	112.1 (12)	118.0 (8)	0.34
Eye–hand coordination	107.5 (16)	101.5 (7)	0.38
Performance (visual–spatial)	101.5 (15)	90 (4.7)	0.06
General Griffiths	104.6 (11)	103.3 (3)	0.84

PMTCT, prevention of mother-to-child transmission.

**TABLE 2-A1:** Comparison of participant characteristics for those who had detectable and undetectable viral loads at Griffiths mental development scales testing reported as mean (standard deviation).

Viral load at testing	Detectable VL *n* = 9	Undetectable VL *n* = 20	*p*
Birth weight	2912.2 (576)	3042.5 (475)	0.37
Baseline VL copies	45105 (70493) (*n* = 8)	16000 (318345) (*n* = 18)	0.36
ART start days from birth	9.3 (6.4)	5.8 (3.8)	0.14
CD4 closest to GMDS Absolute count CD4%	2061.2 (1035) 30.8 (2.7)	2180.8 (560) 35.4 (8.1)	0.25 0.21
CD8 closest to GMDS Absolute count CD8%	2348.1 (2092) 30.2 (8.7)	1723.7 (948) 26.3 (10.5)	0.56 0.26
CD4/CD8 ratio closest to GMDS	1.2 (0.6)	1.6 (0.8)	0.21
CD4%/CD8% ratio closest to GMDS	1.15 (0.6)	1.6 (0.9)	0.17
CD4 baseline Absolute count CD4%	2040.6 (816) 40.0 (12.4)	2099.6 (818) 47.1 (14.4)	0.69 0.26
Growth: WHO *z*-scores for age Weight Head circumference Length	−0.0 (1.1) 0.37 (0.8) −1.02 (1.1)	−0.13 (0.8) 0.17 (1.1) −1.13 (1.0)	0.87 0.52 0.71

ART, Antiretroviral therapy; GMDS, Griffiths Mental Development Scale; VL, viral load.

**TABLE 3-A1:** Summary of scores on the Griffiths mental development scales locomotor and General Griffiths from controls on South African studies at similar ages.

Author/study	Age	Locomotor	General Griffiths
Perez: non-anaemic controls^34^	9 months	136	127.0
Davies: non-foetal alcohol syndrome controls^33^	7–12 months	100	104.0
Laughton: HIV exposed uninfected controls^10^	11 months	102	107.0
Current study	11 months	95.9	103.6
Amod: South African sample^29^	13–16 months	98	102.0
Springer: HIV exposed uninfected controls^32^	17–19 months	87	87.0
